# Factors Influencing Web-Based Survey Response for a Longitudinal Cohort of Young Women Born Between 1989 and 1995

**DOI:** 10.2196/11286

**Published:** 2019-03-25

**Authors:** Deborah Loxton, Melissa L Harris, Peta Forder, Jennifer Powers, Natalie Townsend, Julie Byles, Gita Mishra

**Affiliations:** 1 Research Centre for Generational Health and Ageing Faculty of Health and Medicine University of Newcastle Callaghan Australia; 2 Institute for Social Science Research Faculty of Humanities and Social Sciences The University of Queensland Herston Australia

**Keywords:** retention, attrition, young adult, Web-based survey, social media, women’s health

## Abstract

**Background:**

With health research practices shifting toward rapid recruitment of samples through the use of online approaches, little is known about the impact of these recruitment methods on continued participation in cohort studies.

**Objective:**

This study aimed to report on the retention of a cohort of young women who were recruited using an open recruitment strategy.

**Methods:**

Women from the 1989-95 cohort of the Australian Longitudinal Study on Women’s Health, recruited in 2012 and 2013 were followed up annually via Web-based surveys in 2014, 2015, and 2016. Prevalence ratios for survey response were calculated using log-binomial models with generalized estimating equations including demographic, health-related, and recruitment method characteristics examined as explanatory factors.

**Results:**

Of the 17,012 women who completed the baseline survey (Survey 1) in 2012 to 2013, approximately two-thirds completed Survey 2 (2014), and just over half completed Surveys 3 (2015) and 4 (2016). Women demonstrated transient patterns of responding with 38.21% (6501/17,012) of women completing all 4 surveys. Although retention of young women was associated with older age, higher education, higher self-rated health status, and low engagement with adverse health behaviors, the method of recruitment was a key determinant of study participation in the multivariate model. Although women were more likely to be recruited into the cohort via social media (eg, Facebook), retention over time was higher for women recruited through traditional media and referral approaches.

**Conclusions:**

A balance must be obtained between achieving representativeness, achieving rapid cohort recruitment, and mitigating the pitfalls of attrition based on recruitment method in the new era of cohort studies, where traditional recruitment methods are no longer exclusively viable options.

## Introduction

Participation in epidemiologic studies has been declining over the past 30 years. This has been attributed to declining volunteerism, the proliferation of market research studies, perceived irrelevance, and increased demands on participant involvement [1]. Together with the changing technological landscape and shift toward digital communication practices, this has led to innovative approaches to participant recruitment that take advantage of online recruitment through social media advertising and blog-seeding [2-4]. Although these techniques might be successful in recruiting participants to a single study [2,5], little is known about the impact of these recruitment techniques on participant retention in cohort studies.

A review of the current literature revealed consistent associations between sociodemographic and health factors and attrition. In conventional cohort studies using paper-based surveys, attrition (permanent or temporary loss to follow-up) is more likely to occur if participants are younger [6,7], less educated [8,9], have poorer health [6,7,10], or are smokers [6,11]. Retention (active participation at a survey wave) within conventional longitudinal studies among younger people has been credited to ongoing contact with participants, via such avenues as regular postcards and phone calls [12-14]. As internet-based research increases, it is important to understand what leads participants to return to a Web-based survey and what biases might be introduced by the disparities between those who return and those who do not, particularly for young people who are notoriously hard to engage in longitudinal health research [1,2]. It is also important to understand the influence of sociodemographic factors on recruitment method and the joint impact on retention. For example, we found that recruitment method differed by age [3] and, on the basis of past research, we would expect age to also influence retention [6,7].

The aim of this study was to examine factors that influence retention in a longitudinal cohort study of young women who were openly recruited through the internet and social media, as well as traditional media recruitment methods [3]. Specifically, we wished to identify the characteristics associated with retention, with a particular focus on whether retention was influenced by different recruitment methods. On the basis of past research, we hypothesized that women who were younger [6,7], had lower levels of education [8,9], had relatively poorer health [6,7,10], and who used tobacco [6,11] would be less likely to continue completing surveys compared with other women. We further hypothesized that the recruitment method may influence retention and that some of this influence may be due to age [3].

## Methods

### Overview of the Study Design

This study included data from the 1989-95 cohort of the Australian Longitudinal Study on Women’s Health (ALSWH), a national population-based health study of Australian women. Details regarding the recruitment strategy for this cohort are described in detail elsewhere [3]. Briefly, however, between 2012 and 2013, women born between 1989 and 1995 (aged 18 to 23 years) were recruited using an open recruitment method that relied on both social media as well as traditional recruitment strategies. Additional eligibility for study inclusion included living in Australia, possessing a Medicare (ie, Australia’s national health insurer) number (Australian and New Zealand citizens and permanent residents living in Australia are eligible for a Medicare number), and consenting to have survey data linked with administrative data (eg, records of health service use). Verification for data linkage was conducted by the Australian Department of Human Services matching participants on name, address, date of birth, and Medicare number.

Unlike the previous ALSWH cohorts who were recruited through the Medicare database, recent recruitment of young women demonstrated that this approach was no longer a viable option for the recruitment of young women for the purpose of conducting health surveys [15]. Recruitment strategies for the new cohort of women aged 18 to 23 years included paid Facebook advertising, promotion using social and other internet-based media (eg, forum posts, Gumtree advertisements, and Twitter), paid and unpaid promotion through traditional media (eg, face-to-face events, posters, print, and radio media promotion), incentives (eg, chance to win a AU $50 gift voucher or fashion promotion which entailed a chance to win an exclusive pair of Black milk tights), and peer referral. Owing to the slow rate of recruitment at the beginning, the campaign comprised 2 promotions: the first delivered by ALSWH (October 2012 to December 2013) and the second by a marketing company under the branding of Women’s Health of Australia! (October 2013 to December 2013). The slogans, branding, and incentives offered under each of the promotions are described in detail elsewhere [3].

The 17,012 women who responded to the open recruitment invitation and completed the Web-based survey were found to be broadly representative of similarly aged Australian women in terms of demographics, with some over-representation of more educated women [5]. Unlike the original 3 ALSWH cohorts who completed surveys on a 3-year rolling schedule, the newest cohort has completed yearly surveys.

To meet the aims of the study, surveys were focused on demographic, economic and social factors, health behaviors, self-reported anthropometric measures, physical and mental health, and health service use. To reduce participant burden, not all questions are asked at every survey, with a core set of items included and themed items included in every second or third survey (eg, complementary and alternative therapies are included in Survey 2 (measured in 2014), whereas the additional theme for Survey 3 (measured in 2015) was on parental socioeconomic position and adverse childhood experiences) [16].

### Participants

Data for this study were obtained from the ALSWH 1989-95 cohort who completed surveys in 2012 to 2013 (Survey 1), 2014 (Survey 2), and 2015 (Survey 3).

### Measures

Response status: The women were resurveyed annually via Web-based surveys in 2014, 2015, and 2016. Women were classified as respondents to a particular survey if they provided answers to at least 5 items from that survey. The number of questions varied between 56 and 113 across the 3 follow-up surveys.

Demographic variables at Survey 1 included age and area of residence. Area of residence was based on the Accessibility/Remoteness Index of Australia Plus index of distance from the nearest urban center (major cities; inner regional; and outer regional, remote, or very remote) [17]. The highest level of education completed was classified as (1) less than 12 years of schooling, (2) Grade 12 or equivalent, (3) certificate or diploma, and (4) university. Student and employment status were each classified as not studying or not employed and part‑time or full-time. The ability to manage with available income was reported as easy, not too bad, difficult some of the time, difficult all the time, or impossible. Relationship status was classified as married; living in a de facto relationship; or not married or partnered.

Health-related characteristics at Survey 1 included self-rated health, psychological distress, health behaviors, body mass index (BMI), physical activity, and experience of partner violence. Self‐rated health was measured by the general health item, “In general would you say your health is:” with response options of excellent; very good; good; fair; or poor [18]. Psychological distress was measured by the Kessler Psychological Distress Scale (K10) [19], which comprises 10 items that measure recent depression and anxiety. The summed responses to these items were categorized to indicate low, moderate, high, and very high levels of psychological distress [20]. Smoking was classified into never smoker, ex‑smoker, and current smoker. Alcohol consumption was based on the level of risk identified according to the 2009 guidelines [21] with episodic risk defined as drinking 5 or more standard drinks on 1 occasion and long-term risk as drinking more than 2 drinks per day on average. Using these definitions, 4 categories of alcohol consumption were used: (1) no risk; (2) low long-term risk, low episodic risk; (3) low long-term risk, high episodic risk; and (4) high long-term risk, irrespective of episodic risk. Marijuana use and other illicit drug use were categorized according to most recent use: never; recent use (in the last 12 months); past use (more than 12 months ago); recent and past use. BMI (kg/m^2^) was categorized according to World Health Organization guidelines as underweight (BMI less than 18.5), healthy weight (BMI 18.5 to 24.9), overweight (BMI 25 to 29.9), and obese (BMI 30 or more) [22]. Physical activity level was based upon the frequency and duration of leisure-time activity in the last week and classified as sedentary, low, moderate, or high [23]. Partner violence was an affirmative response to the item: “Have you ever been in a violent relationship with a partner or spouse?”

Recruitment method: When women were first recruited into the 1989-95 cohort, an item in the baseline survey asked them what had led them to take part in the study. Responses were grouped into 5 broad recruitment methods: (1) Facebook, including posts on the ALSWH pages and paid advertising; (2) other online media (eg, Twitter, Tumblr, and blogs); (3) referral (eg, direct contact from ALSWH staff, referral from professional and personal networks, and existing ALSWH participants); (4) traditional media (eg, television, radio, and magazine advertising); and (5) a fashion promotion, which involved an incentive of the chance to win a pair of exclusive tights [3].

### Analyses

Baseline characteristics at Survey 1, including mode of recruitment, are presented according to participants who responded to follow-up surveys (respondents) versus participants who did not (nonrespondents). Response rates across the first 3 follow-up surveys are presented in a multimedia appendix according to pattern of response, demographic, and health-related characteristics as well as recruitment method.

Prevalence ratios (PR) for survey response were calculated using a log-binomial model with generalized estimating equations to account for the correlation of repeated observations across surveys. A total of 3 models were investigated incorporating explanatory variables measured at baseline: (1) survey; (2) survey and recruitment method; and (3) survey, recruitment method, selected demographic, and health‑related variables. An interaction between age and recruitment method was also investigated. Results were deemed to be statistically significant if *P*<.01, and analyses were conducted using SAS Software 9.4 (TS1M3) for Windows (SAS Institute Inc, Cary, NC, USA).

## Results

In 2012 to 2013, 17,012 women completed Survey 1 (baseline). Surveys were conducted annually thereafter, with 66.68% (11,344/17,012) of women completing the second survey in 2014, 52.67% (8961/17,012) of women completing the third survey in 2015, and 52.94% (9007/17,012) of women completing the fourth survey in 2016. All 3 follow-up surveys were completed by 38.21% (6501/17,012) of women, whereas 19.54% (3324/17,012) of women completed 2 follow-up surveys, 18.58% (3161/17,012) of women completed 1 follow-up survey only, and 23.67% (4026/17,012) women did not respond to any of the follow-up surveys. The frequency of response patterns across 3 follow-up surveys is presented in Table 1.

Women who responded at subsequent surveys (2, 3, or 4) were more likely to be older at baseline, reported better health, and were more likely to manage on their available income at baseline, whereas women who did not respond again after Survey 1 were more likely to report smoking, drinking, or other drug use at baseline, be sedentary, or report experiencing domestic violence (see Multimedia Appendix 1). Responders were more likely to have been recruited via referral or traditional media methods, whereas nonrespondents were more likely to report being recruited through Facebook and other social media strategies.

Response rates across the first 3 follow-up surveys are presented in Multimedia Appendix 2.

**Table 1:** Frequency of response patterns across 3 follow-up surveys (Survey 2, Survey 3, and Survey 4) for the women who completed Survey 1 (N=17,012).

Pattern of response to follow-up surveys			Statistics, n (%)
0 follow-up surveys			4026 (23.67)
**1 follow-up survey only**			3161 (18.58)
	Survey 2 only	2089 (12.28)	
	Survey 3 only	474 (2.79)	
	Survey 4 only	598 (3.52)	
**2 follow-up surveys**			33324 (19.54)
	Survey 2 and Survey 3	1416 (8.32)	
	Survey 2 and Survey 4	1338 (7.87)	
	Survey 3 and Survey 4	570 (3.35)	
**3 follow-up surveys**			6501 (38.21)
	Survey 2, Survey 3, and Survey 4	6501 (38.21)	

Participants were 20% less likely to respond after the first follow-up survey (95% CI 0.79-0.80, see Table 2). Compared with women who were recruited via Facebook, women who were recruited via referral or traditional media were more likely to respond at subsequent surveys (PR=1.11; 95% CI 1.07-1.15 and PR=1.18; 95% CI 1.14-1.22, respectively), whereas some recruited via the fashion promotion strategy were slightly less likely to respond (PR=0.94; 95% CI 0.91-0.97). Women were less likely to respond if they had not completed Grade 12 at baseline (PR=0.80; 95% CI 0.76-0.84), had extreme difficulties managing with their available income (PR=0.95; 95% CI 0.93-0.97), were smokers (PR=0.82; 95% CI 0.79-0.85), or had very high levels of psychological distress (PR=0.96; 95% CI 0.93-0.98). There was no evidence of an interaction between age and recruitment method (*P*>.01, data not shown).

**Table 2:** Factors associated with survey response over 3 follow-up surveys using baseline (Survey 1) characteristics as explanatory variables (N=17,012; 187 participants were excluded due to missing data). The included explanatory factors for predicting response at follow-up surveys were model 1 (survey), model 2 (survey + recruitment method), and model 3 (survey + recruitment method + other characteristics; eg, selected demographic/health factors).

Baseline characteristics		Model 1		Model 2		Model 3	
		PR (95% CI)^a^	*P* value	PR (95% CI)	*P* value	PR (95% CI)	*P* value
**Survey**							
	2 (2014)	1	—^b^	1	—	1	—
	3 (2015)	0.79 (0.78-0.80)	<.001	0.79, (0.78-0.80)	<.001	0.80 (0.79-0.81)	<.001
	4 (2016)	0.79 (0.78-0.80)	<.001	0.80 (0.79-0.81)	<.001	0.80 (0.79-0.81)	<.001
**Recruitment method**							
	Facebook	—	—	1	—	1	—
	Other social media	—	—	1.06 (1.01-1.11)	.0114	1.04 (0.99-1.08)	.10
	Referral	—	—	1.14 (1.11-1.18)	<.001	1.11 (1.07-1.15)	<.001
	Traditional media	—	—	1.22 (1.18-1.26)	<.001	1.18 (1.14-1.22)	<.001
	Fashion promotion	—	—	0.97 (0.94-1.00)	.0321	0.94 (0.91-0.97)	<.001
**Baseline age**							
	18-20 years	—	—	—	—	1	—
	21-23 years	—	—	—	—	1.08 (1.06-1.10)	<.001
**Area of residence**							
	Major cities	—	—	—	—	1	—
	Inner regional	—	—	—	—	1.01 (0.98-1.03)	.59
	Outer regional and remote areas	—	—	—	—	0.97 (0.93-1.01)	.11
**Education**							
	Grade 12 or more	—	—	—	—	1	—
	Less than Grade 12	—	—	—	—	0.80 (0.76-0.84)	<.001
**Income management**							
	Easy/not too bad/difficult sometimes	—	—	—	—	1	—
	Impossible/difficult all the time	—	—	—	—	0.95 (0.93-0.97)	<.001
**Obesity**							
	Not obese (BMI^c^<30)	—	—	—	—	1	—
	Obese (BMI ≥ 30 or more)	—	—	—	—	0.95 (0.92-0.98)	.0035
**Smoking status**							
	Nonsmoker/exsmoker	—	—	—	—	1	—
	Current smoker	—	—	—	—	0.82 (0.79-0.85)	<.001
**Self-rated health**							
	Excellent/very good/good	—	—	—	—	1	—
	Fair/poor	—	—	—	—	0.99 (0.96-1.02)	.61
**Psychological distress**							
	Not very high (K10 score<30)	—	—	—	—	1	—
	Very high distress (K10 score ≥30)	—	—	—	—	0.96 (0.93-0.98)	.0016

^a^PR (95% CI): prevalence ratio with 95% CI.

^b^Not applicable.

^c^BMI: body mass index.

## Discussion

### Principal Findings

This study aimed to provide an understanding of what factors influence longitudinal study retention rates for young people recruited through an open recruitment strategy. Importantly, similar to the ALSWH 1973-78 cohort, this new cohort of young women demonstrated generational patterns in responding, with younger women more likely to be transient in their participation over time. On the basis of previous research utilizing traditional recruitment methods [7], we also hypothesized that women who had lower levels of education were obese and those who smoked would be less likely to be retained at follow-up surveys compared with other women. Although the findings support these hypotheses, we found that the method of recruitment was associated with ongoing survey response, even after controlling for sociodemographic and health-related characteristics. This is a novel finding in the new era of online recruitment.

### Comparison With Prior Work

Although relatively new as a recruitment tool in health research, social media has previously been lauded for its cost-effectiveness, increased reach of participants (including those that are hard to reach and minority populations), flexibility, and ability to provide largely representative samples [2,5,24]. Here, we were able to extend our knowledge regarding the impact of recruitment method on cohort retention. The majority of women were successfully recruited into the cohort via Facebook or other online avenues, with 65% to 69% of these women returning to complete the first follow-up survey. This is consistent with cohort retention rates using traditional random sampling recruitment strategies [25]. Particularly, retention among similarly aged women recruited into the ALSWH in 1996 demonstrated a similar pattern, with 68% of participants retained at the second survey (conducted 4 years later), with participant attrition stabilizing over time. Importantly, for the newest 1989-95 ALSWH cohort of young women, a part of the study design included shorter surveys to be conducted annually in an effort to mitigate the attrition seen in the previous cohort of younger women who were administered surveys on a 3-year rolling schedule. However, this strategy did not appear to be effective in mitigating the attrition between Surveys 1 and 2, with an overall response rate of 67% just 12 months later. The main reason for nonresponse was that they could not be contacted, which was the same reason as that found for the 1973-78 cohort [25]. It is important to note, however, that although attrition was the highest between Surveys 1 and 2, this effect had plateaued by Survey 4.

Although a smaller number of participants were recruited through traditional media and referral avenues, the retention rate for this group of women remained higher than those recruited through social media. This finding has important implications for online recruitment particularly for young women. It is posited that recruitment through Facebook and other social media provided an immediate opportunity to click directly through to the first survey, potentially reducing the time to consider the ongoing commitment for participation and also involving less contact with the research team. This is supported by Frandsen et al [26] who argued that the use of social media does not lead to better informed and potentially self-screened participants compared with traditional media. Participants recruited through traditional media and referral have to consciously decide to do the survey and then access it on the web, possibly indicating more thought about completing the first survey and a better understanding that there would be additional questionnaires to follow. In addition, traditional media and referral both provide contact with the research team, either through interviews in traditional media or personal contact through referral. As a result, researchers using social media, such as Facebook, for the purposes of recruitment into longitudinal cohort studies need to consider the amount of participant information that is collected to enable accurate tracking of participants to minimize participant attrition.

Although labor intensive, previous research has identified that implementing multiple methods of retention increased rates by 70% [27]. With reduced initial contact with participants recruited through social media, more personalized methods of follow-up (eg, mail, email, short message service, and telephone) may be even more important to reduce attrition. Therefore, when recruiting a cohort for the purposes of longitudinal research, a balance between achieving representativeness, meeting cohort targets quickly, and understanding potential rates of attrition depending on the method of recruitment is required in the planning stages. Given the fact that traditional random sampling methods are no longer cost-effective to be used exclusively [15], balancing these processes and potential outcomes is particularly important.

Furthermore, the use of incentives is beginning to be highlighted as key factors in the recruitment and retention of young people [27,28]. Importantly, a systematic review found that study retention rates among the 10 studies identified increased with higher monetary values; however, whether cash incentives were more effective than gifts was not clear. This is in contrast with recent findings regarding the use of incentives in relation to cohort recruitment. In particular, an Australian study focused on contraceptive use and pregnancy intentions among similarly aged women found that only small incentives (AU $20 gift card) were required to recruit their demographically representative cohort [2]. In addition, a UK-based cohort study that used a combination of prize draw and gift voucher incentives found that the withdrawal rate was not influenced by whether an incentive was received [29]. In the ALSWH, incentives differed at each survey.

Interestingly, results for health and health behavior factors are in agreement with findings from the 4 ALSWH cohorts born between 1921 and 1926, 1946 and 1951, and 1973 and 1978 [30]. It appears to be that poor health and adverse health behaviors deter ongoing participation across generations and survey modalities. These findings reflect international literature that has demonstrated the impact of health and health behavior on participation in longitudinal health research [11,31-33]. Despite the advent of online recruitment approaches and Web-based surveying and advances in technology that support participant tracking [16], the issue of biases in retention remain.

### Limitations

This study must be considered in light of its limitations. First, we examined rates of attrition among young women. Younger women, particularly in the ALSWH, have been found to be more mobile than women who are older [25]. In addition, these women were born in an age of technological advancement, particularly regarding online and social media. As a result, reasons for attrition may differ by age. It is also important to note that we had a restrictive 5-year age range for our cohort (ie, 18 to 23), which prevented us from being able to understand the nuances associated with age on recruitment strategy. This would require further examination with a broader age range. Second, there is the potential that unmeasured factors may have influenced rates of retention. Finally, although we examined associations between variables, causation cannot be implied.

In addition, the method of recruitment was determined via participant self-report with participants being only able to select 1 mode of invitation. There is the potential that participants were exposed to multiple approaches before joining the study [3] and that they selected the method that resonated the most. Future research is required to assess the number of strategies that are encountered before a commitment to study participation is made. Despite these limitations, the study is balanced by a number of strengths including being a large cohort, which is broadly representative of similarly aged Australians in the general population [3]. Such a cohort allows us to predict the subgroups of women that potentially should be targeted from the outset to remain in the study.

### Conclusions

Although there is a need for more research into the factors that engage participants to commit to a cohort study and to follow through on that commitment, this study offers some insights. In particular, the mode of recruitment appears to create differing levels of commitment, perhaps reflecting the level of personal contact with the research team and time taken to consider enrolling in a study. In addition, demographic and health factors also impact on retention, suggesting that strategies to particularly re-engage those with poor health, adverse health behaviors, and from lower socioeconomic groups may be warranted. Perhaps cohort researchers would benefit from taking a leaf from qualitative methods and undertaking purposive re-engagement, along the lines of purposive sampling where a particular group is targeted for inclusion or, in this case, reinclusion. The more representative a cohort is and the less attrition it has, the more useful its results. However, a balance must be obtained between achieving representativeness, achieving rapid cohort recruitment, and mitigating the pitfalls of attrition based on recruitment method in the new era of cohort studies, where traditional recruitment methods are no longer exclusively viable options.

## Appendix

Multimedia Appendix 1 Baseline characteristics at Survey 1 for women aged 18 to 23 years in 2012 to 2013, according to whether they responded at subsequent surveys.

Multimedia Appendix 2 Response rates of original sample (N=17,012) at follow-up surveys, according to baseline characteristics.

## Abbreviations

ALSWH: Australian Longitudinal Study on Women’s Health
BMI: body mass index
K10: Kessler Psychological Distress Scale
PR: prevalence ratio
