# Supplementary material for: Factors Influencing Web-Based Survey Response for a Longitudinal Cohort of Young Women Born Between 1989 and 1995
Source: J Med Internet Res. 2019 Mar 25;21(3):e11286. doi: 10.2196/11286 (PMC6452283; doi:10.2196/11286)
Supplement: Multimedia Appendix 1 [file jmir_v21i3e11286_app1.pdf]

Multimedia Appendix

Multimedia Appendix 1: Baseline characteristics at Survey 1 for women aged 18 to 23 years in 2012 to 2013, according to whether they responded at subsequent surveys

Baseline characteristic (Survey 1)		All women at Survey 1 (N=17,012), n (%)	Responders at subsequent surveys (N=12,986), n (%)	Nonresponders after Survey 1 (N=4026), n (%)
**Age (at Survey 1)**				
	18-20 years	8493 (49.9)	6261 (48.2)	2232 (55.4)
	21-23 years	8519 (50.1)	6725 (51.8)	1794 (44.6)
**Area of residence**				
	Missing	12	6	6
	Major cities (and overseas)	12,789 (75.2)	9806 (75.5)	2983 (74.2)
	Inner regional	2894 (17.0)	2185 (16.8)	709 (17.6)
	Outer regional, remote, very remote	1317 (7.75)	989 (7.62)	328 (8.16)
**Level of education**				
	Missing	183	109	74
	Less than 12 years schooling	1265 (7.52)	795 (6.17)	470 (11.9)
	Grade 12 or equivalent	7314 (43.5)	5679 (44.1)	1635 (41.4)
	Certificate/Diploma	4416 (26.2)	3255 (25.3)	1161 (29.4)
	University	3834 (22.8)	3148 (24.4)	686 (17.4)
**Student status**				

	Missing	199	121	78
	Not studying	5706 (33.9)	4132 (32.1)	1574 (39.9)
	Part-time study	9398 (55.9)	7292 (56.7)	2106 (53.3)
	Full-time study	1709 (10.2)	1441 (11.2)	268 (6.79)
**Employment**				
	Missing	185	111	74
	Not working	3710 (22.0)	2817 (21.9)	893 (22.6)
	Part-time work	9203 (54.7)	7103 (55.2)	2100 (53.1)
	Full-time work	3914 (23.3)	2955 (23.0)	959 (24.3)
**Ability to manage available income**				
	Missing	188	113	75
	Impossible	734 (4.36)	502 (3.90)	232 (5.87)
	Difficult most of the time	3529 (21.0)	2603 (20.2)	926 (23.4)
	Difficult some of the time	6000 (35.7)	4608 (35.8)	1392 (35.2)
	Not too bad	4773 (28.4)	3724 (28.9)	1049 (26.6)
	Easy	1788 (10.6)	1436 (11.2)	352 (8.91)
**Marital status**				
	Missing	183	109	74
	Married	504 (2.99)	402 (3.12)	102 (2.58)
	De facto	3310 (19.7)	2481 (19.3)	829 (21.0)
	Not married/not in a relationship	13,015 (77.3)	9994 (77.6)	3021 (76.4)
**Recruitment method**				

	Missing	245	112	75
	Facebook	11,771 (70.0)	8851 (68.8)	2920 (73.9)
	Other social media	824 (4.90)	646 (5.02)	178 (4.51)
	Referral	1187 (7.05)	981 (7.62)	206 (5.21)
	Traditional media	910 (5.41)	792 (6.15)	118 (2.99)
	Fashion promotion	2133 (12.7)	1604 (12.5)	529 (13.4)
**Self-rated health**				
	Missing	164	94	70
	Excellent	1091 (6.48)	889 (6.90)	202 (5.11)
	Very good	6062 (36.0)	4768 (37.0)	1294 (32.7)
	Good	6845 (40.6)	5140 (39.9)	1705 (43.1)
	Fair	2373 (14.1)	1755 (13.6)	618 (15.6)
	Poor	477 (2.83)	340 (2.64)	137 (3.46)
**Psychological distress (K10 Score)**				
	Missing	172	103	69
	Low (10-15)	3522 (20.9)	2767 (21.5)	755 (19.1)
	Moderate (16-21)	4976 (29.5)	3907 (30.3)	1069 (27.0)
	High (22-29)	4614 (27.4)	3514 (27.3)	1100 (27.8)
	Very high (30-50)	3728 (22.1)	2695 (20.9)	1033 (26.1)
**Smoking status**				
	Missing	166	97	69
	Non-smoker	10,595 (62.9)	8406 (65.2)	2189 (55.3)
	Ex-smoker	3073 (18.2)	2351 (18.2)	722 (18.2)
	Current smoker	3178 (18.9)	2132 (16.5)	1046 (26.4)

**Pattern of alcohol consumption**				
	Missing	166	97	69
	No risk	1266 (7.52)	970 (7.53)	296 (7.48)
	Low long-term risk, low episodic risk	13,094 (77.7)	10,119 (78.5)	2975 (75.2)
	Low long-term risk, high episodic risk	1863 (11.1)	1376 (10.7)	487 (12.3)
	High long-term risk, high episodic risk	623 (3.70)	424 (3.29)	199 (5.03)
**BMI (kg/m^3)**				
	Missing	735	650	85
	Underweight (BMI<18.5)	1285 (7.89)	976 (7.91)	309 (7.84)
	Acceptable (BMI 18.5-24.9)	9711 (59.7)	7474 (60.6)	2237 (56.8)
	Overweight (BMI 25.0-29.9)	3122 (19.2)	2350 (19.0)	772 (19.6)
	Obese (BMI>=30)	2159 (13.3)	1536 (12.5)	623 (15.8)
**Physical activity level**				
	Missing	159	94	65
	Sedentary/no exercise	1031 (6.12)	741 (5.75)	290 (7.32)
	Low	4111 (24.4)	3141 (24.4)	970 (24.5)
	Moderate	3583 (21.3)	2823 (21.9)	760 (19.2)
	High	8128 (48.2)	6187 (48.0)	1941 (49.0)
**Marijuana use**				
	Missing	170	100	70

	Never	7844 (46.6)	6187 (48.0)	1657 (41.9)
	Recent use (<12 months ago)	4545 (27.0)	3286 (25.5)	1259 (31.8)
	Past use (>12 months ago)	3872 (23.0)	2946 (22.9)	926 (23.4)
	Recent and past use	581 (3.45)	467 (3.62)	114 (2.88)
**Other illicit drug use**				
	Missing	172	100	72
	Never	11,976 (71.1)	9370 (72.7)	2606 (65.9)
	Recent use (<12 months ago)	2657 (15.8)	1881 (14.6)	776 (19.6)
	Past use (>12 months ago)	1921 (11.4)	1414 (11.0)	507 (12.8)
	Recent and past use	286 (1.70)	221 (1.72)	65 (1.64)
**Partner/spouse violence**				
	Missing	422	291	131
	Never experienced	14,408 (86.8)	11,155 (87.9)	3253 (83.5)
	Experienced	2182 (13.2)	1540 (12.1)	642 (16.5)

Note: percentages apply to non-missing responses only

This is a Multimedia Appendix to a full manuscript published in the J Med Internet Res. For full copyright and citation information see http://dx.doi.org/10.2196/jmir.11286
