# Supplementary material for: Factors Influencing Web-Based Survey Response for a Longitudinal Cohort of Young Women Born Between 1989 and 1995
Source: J Med Internet Res. 2019 Mar 25;21(3):e11286. doi: 10.2196/11286 (PMC6452283; doi:10.2196/11286)
Supplement: Multimedia Appendix 2 [file jmir_v21i3e11286_app2.pdf]

Multimedia Appendix

Multimedia Appendix 2: Response rates of original sample (N=17,012) at follow-up surveys, according to baseline characteristics

Baseline characteristic (Survey 1)		Response Rate (%) Survey 2 (2014) N=11,344	Survey 3 (2015) N=8,961	Survey 4 (2016) N=9,007
Age	18-20 years	64.3	49.7	49.7
	21-23 years	69.0	55.7	56.1
Area of residence	Major cities (& overseas)	66.9	53.2	53.2
	Inner regional	66.4	51.0	52.5
	Outer regional, remote & very remote	65.1	51.2	51.2
Education	Less than 12 years schooling	52.4	38.7	35.9
	Grade 12 or equivalent	68.7	54.0	54.8
	Certificate/Diploma	63.6	48.9	47.6
	University	72.1	60.1	61.8
Student status	Not studying	62.6	46.9	46.4
	Part-time study	68.0	54.4	55.3
	Full-time study	75.6	64.5	63.7
Employment	Not working	67.3	51.7	50.7
	Part-time work	67.8	54.0	54.6
	Full-time work	64.6	51.3	51.9
Ability to manage on available income	Impossible	60.5	46.5	41.6
	Difficult most of the time	63.5	48.9	47.6
	Difficult some of the time	67.0	52.4	52.8
	Not too bad	69.0	55.2	57.1
	Easy	70.5	58.7	59.3
Marital status	Married	71.0	56.5	57.1

	De facto	64.8	50.3	50.7
	Not married or partnered	67.3	53.4	53.6
Recruitment method	Facebook	65.3	52.1	52.3
	Other social media	69.5	54.7	54.6
	Referral	73.9	61.4	61.9
	Traditional media	78.9	64.2	66.4
	Fashion promotion	65.7	47.0	46.6
Self-rated health	Excellent	71.0	59.5	63.1
	Very good	69.0	56.4	57.1
	Good	65.8	50.3	50.5
	Fair	63.9	49.2	47.0
	Poor	62.3	48.6	47.4
Psychological distress (K10 Score)	Low (10-15)	70.1	55.9	57.7
	Moderate (16-21)	68.5	56.8	56.9
	High (22-29)	66.5	51.3	51.5
	Very high (30-50)	62.4	46.7	45.7
Smoking status	Non-smoker	70.1	56.8	57.4
	Ex-smoker	67.2	51.4	51.4
	Current smoker	56.1	41.3	40.6
Pattern of alcohol consumption	No risk	67.4	52.2	52.1
	Low long-term risk, low episodic risk	68.1	53.8	54.0
	Low long-term risk, high episodic risk	61.3	49.6	50.2
	High long-term risk, high episodic risk	57.3	45.4	43.7
Body Mass Index (kg/m^2)	Underweight (BMI<18.5)	67.6	53.9	52.7
	Acceptable (BMI 18.5-24.9)	67.4	54.3	55.2
	Overweight (BMI 25.0-29.9)	65.9	53.2	52.0

	Obese (BMI>=30)	63.2	49.1	47.0
Physical activity level	Sedentary/no exercise	62.4	46.9	45.4
	Low	67.5	52.8	52.8
	Moderate	68.7	54.9	55.5
	High	66.4	52.8	53.2
Marijuana use	Never	69.9	56.3	56.9
	Recent use (<12 months ago)	62.0	48.0	48.0
	Past use (>12 months ago)	66.3	51.0	51.1
	Recent and past use	69.5	57.1	55.1
Other illicit drug use	Never	69.1	55.1	55.5
	Recent use (<12 months ago)	59.9	45.7	45.4
	Past use (>12 months ago)	63.1	48.8	48.9
	Recent and past use	66.4	53.5	52.1
Partner/spouse violence	Never experienced	68.1	54.2	54.6
	Experienced	59.4	44.6	43.6

This is a Multimedia Appendix to a full manuscript published in the J Med Internet Res. For full copyright and citation information see http://dx.doi.org/10.2196/jmir.11286
